# Candidate gene mutations of patients with astrocytoma who present with seizures: evidence from whole exome sequencing

**DOI:** 10.3389/fonc.2025.1577344

**Published:** 2025-07-11

**Authors:** Kanitpong Phabphal, Pongsakorn Choochuen, Anukoon Kaewborisutsakul, Thara Tunthanathip, Surasak Sangkhathat

**Affiliations:** ^1^ Neurology Unit, Department of Medicine, Faculty of Medicine, Prince of Songkla University, Songkhla, Thailand; ^2^ Department of Biomedical Sciences and Biomedical Engineering, Prince of Songkla University, Songkhla, Thailand; ^3^ Translational Medicine Research Center, Faculty of Medicine, Prince of Songkla University, Songkhla, Thailand; ^4^ Neurological Surgery Unit, Department of Surgery, Faculty of Medicine, Prince of Songkla University, Songkhla, Thailand; ^5^ Department of Surgery, Faculty of Medicine, Prince of Songkla University, Songkhla, Thailand

**Keywords:** astrocytoma, seizure, NMDA, IDH1, glutamate

## Abstract

Epileptic seizures are common and substantially impact long-term quality of life. However, the risk factors for preoperative seizures among patients with astrocytomas remain unclear. This study aimed to identify the candidate genes associated with seizure before operation among patients with astrocytomas. We conducted a single-center cohort study including 34 patients with astrocytomas that presented with or without preoperative seizures and analyzed differential gene expression, evaluating a total of 142 candidate genes, selected based on a literature review, and categorized into functional groups (e.g., glutamatergic, oncogenic, chromatin-modifying). Clinical characteristics, including age, sex, tumor location, grade, and size, and peritumoral edema, were similar between the seizure and non-seizure groups. Glutamate receptor mutations were identified in 15% of the non-seizure group and 78.6% of the seizure group. Patients with astrocytomas who presented with seizures had significantly more mutations in glutamate-related genes, including *NMDA* (64.3% vs. 20%, p = 0.01) and *IDH1* (42.7% vs. 10%, p = 0.04). Group III metabotropic glutamate receptor alterations were found in 1 patient in the non-seizure group (n = 19) and in 5 patients in the seizure group (n = 9) (p = 0.06). No significant differences were observed in other glutamate receptors subtypes or related genes. In conclusion, seizures in patients with astrocytomas are associated with *IDH1* and *NMDA* receptor mutations, rather than other clinicopathological factors or other glutamate-related genes. Future research should involve larger multicenter studies and conduct a functional analysis to identify new treatment targets and provide additional evidence to guide clinical decision-making.

## 1 Introduction

Seizures are one of the most common presenting symptoms in patients with gliomas that also decrease their quality of life (1, 2). The prevalence of epilepsy varies depending on patient- and tumor-dependent factors, especially the histological tumor type or genetic mutation. Epilepsy is reported to occur in approximately 75% of patients with low-grade astrocytomas, whereas the reported incidence among patients with glioblastomas and meningiomas is 30–50% and 30–60%, respectively (2). Various molecular mechanisms and pathways have been reported to be involved in the pathophysiology of glioma-associated epilepsy. Gene mutations have been shown to promote seizures in gliomas, especially those involving isocitrate dehydrogenase (*IDH*). Wild-type *IDH1* has been associated with lower seizure incidence compared to *IDH1* mutations in several studies (3–6). A recent retrospective study in patients with glioma demonstrated that preoperative seizures were significantly more frequent in tumors harboring *IDH* mutations (6). This study also demonstrated that the accumulation of 2-hydroxyglutarate (2HG), a metabolite produced by mutant *IDH*, was linked to seizure occurrence, suggesting that metabolic dysregulation may contribute to glioma-related epileptogenesis (6, 7)

The hypothesized that D-2 hydroxyglutarate (D2HG) produced by the *IDH* mutant is like glutamate in terms of chemical structure, which in turn promotes neuronal activity (3, 5). Other gene mutations associated with glutamate overactivity have been reported. Jo et al. conducted a retrospective study in low-grade glioma to evaluate clinical risk factors, *IDH* mutation, and 1p/19q codeletion status and found that male sex was associated with preoperative seizures but not *IDH* mutation or 1p/19q codeletion (8).

Recent studies have evaluated the association between somatic mutations and preoperative seizures. This aberrant activity is mediated by the abnormal expression of various genes including cystine/glutamate antiporter solute carrier family 7-member 11 (xCT), glutamate transporter 1 (GLT-1), and branched-chain amino acid transaminase 1 (BCAT1) (9). In several studies, aberrantly expressed enzymes and proteins in the tumor microenvironment have been shown to promote epilepsy in patients with gliomas by altering the perineuronal environment such as mental retardation X-linked retention, 1p/19q co-deletion, O6-methylguanine-DNA methyltransferase gene promoter methylation, and p533 but inconsistent results (2, 7, 10–13). In the past decade, researchers have identified mutations in more than 70 genes that have been associated with glioma-associated epilepsy (2, 5–13). Advances in next-generation sequencing have facilitated comprehensive tumor genome profiling, providing new insights into the somatic mutations and genomic alterations involved in tumorigenesis. These technologies have also begun to uncover the genetic basis of some clinical manifestations such as tumor-associated seizures.

However, previous studies have focused on gliomas. Different tumor types may play a role in the difference of epileptogenesis (2). Prior studies have evaluated different patient and molecular tumor characteristics. The impact of genetic variation on seizure development has only been characterized in a subset of isolated somatic mutations, and assessments have been conducted for all types of gliomas (9). To date, no study has focused on whole gene mutations in patients with astrocytomas presenting with seizures. Additionally, limited studies have been conducted using tumor tissue whole-exome sequencing to comprehensively analyze the role of gene mutations in patients with astrocytomas who present with seizures. Understanding the role of glutamate in glioma-associated epilepsy is crucial for the development of targeted therapeutic strategies to better manage seizures in patients with tumors. Recent studies have also highlighted how metabolic reprogramming contributes to glioma-related epilepsy (GRE). For example, *IDH1*-mutant glioma cells were shown to release D-2-hydroxyglutarate (D2HG), which promotes lactate accumulation in peritumoral neurons via the mTOR pathway, thereby increasing neuronal hyperexcitability and seizure risk (14). In addition, a recent bioinformatics analysis revealed that synaptic signaling pathways, including glutamatergic, GABAergic, and cholinergic networks, are commonly dysregulated in GRE (15).

Herein, we aimed to identify the candidate genes associated with seizure before operation among patients with astrocytomas.

## 2 Materials and methods

This prospective, observational study was conducted between January 2021 and January 2023. Patients with astrocytomas who presented with or without seizures prior to surgery were included. A total of 34 astrocytoma samples were obtained from the biobank of the Faculty of Medicine, Prince of Songkla University, between May 2022 and June 2023.

### 2.1 Patient grouping and seizure classification

Patients were divided into two groups based on the presence or absence of clinical seizures prior to tumor resection. The seizure group included patients with documented preoperative seizures diagnosed according to the 2017 International League Against Epilepsy criteria (16). The non-seizure group comprised patients with no clinical history of seizures before surgery, as determined by a retrospective review of medical records and neurological consultations. Routine electroencephalography (EEG) was not performed in asymptomatic individuals, in accordance with standard clinical practice at our center.

Data on patient characteristics, including demographics and neuroimaging findings, were collected. Clinical information was obtained from patients’ electronic medical records, including age, sex, seizure type, seizure duration, and Karnofsky Performance Scale (KPS) score. Radiological features evaluated included tumor location, side, and volume. Tumor volume was calculated based on the T2/FLAIR hyperintense areas. Pathological diagnoses were made in accordance with the 2021 WHO classification of central nervous system tumors (17).

### 2.2 Biological samples, sequencing template preparation, and whole-exome sequencing

The DNA was extracted using the DNeasy Blood & Tissue Kit (Qiagen Inc.). The quantity and quality of the extracted DNA were subsequently assessed using Nanodrop (Thermo Fisher Scientific, Inc.) and TapeStation (Agilent Technologies, Inc.). Whole exome sequencing was performed using the Agilent SureSelect XT Human All Exon v8 library preparation system (Agilent Technologies, Inc.). Library quantification was carried out with the Qubit dsDNA High Sensitivity Assay Kit (Invitrogen, Carlsbad, CA, USA), while size measurements were conducted using the Agilent D1000 ScreenTape assay. Sequencing was executed on the Illumina NovaSeq-6000 platform (Illumina, San Diego, California, USA) with paired-end reads of 150 bp. The average targeted coverage depth achieved was 200x.

We assessed the quality of the paired-end sequence files using FastQC (version 0.11.9) and trimmed them with Trimmomatic (version 0.39). The BWA program (version 0.7.17) was employed to align the optimally prepared FASTQ files to the human reference genome (version GRCh38.13). The resulting Sequence Alignment Map (SAM) files were converted to the Binary Alignment Map (BAM) format and sorted using SAMtools (version 1.17) (4). The sorted BAM files were regrouped, and identical sequences were marked using Picard (version 3.0.0). Base quality score recalibration was performed on the unduplicated BAM files with the Genome Analysis Toolkit (GATK, version 4.4.0) to adjust the base quality scores. Variant calling was conducted using Mutect2 in tumor-only mode. A public Panel of Normals (PON) was retrieved from the GATK public repository (https://storage.googleapis.com/gatk-best-practices/somatic-hg38/1000g_pon.hg38.vcf.gz). We utilized GATK4 tools, including GetPileupSummaries, CalculateContamination, and FilterMutectCalls, using default parameter settings to filter the variants identified by Mutect2. The resulting variants were annotated using Funcotator, and the annotated mutational data were stored in MAF files. We utilized the maftools package in R (18) to summarize and visualize the data.

We conducted an extensive literature review to identify genes associated with GRE, emphasizing on glutamatergic signaling and tumorigenic pathways. The final gene panel, categorized into functional groups (e.g., synaptic, oncogenic, chromatin-related), is detailed in **Supplementary Table 1**. A total of 142 genes were selected for somatic mutation screening based on their known involvement in gliomagenesis, glutamatergic signaling, chromatin regulation, and epilepsy-related pathways (see **Supplementary Table 1**) for functional categorization. Also, we had grouped the glutamate receptor genes into four groups: 1) alpha-amino-3-hydroxy-5-methyl-4-isox- azole propionic acid (AMPA) receptors, 2) kainate receptors, 3) NMDA receptors, and 4) metabotropic glutamate receptors. To identify the factors associated with seizures prior to operation, we analyzed clinical characteristics and gene mutations.

All the patients underwent surgical tumor resection at the Songklanagarind Hospital, and the clinical records were reviewed retrospectively. All the tumor samples were collected from a certified institutional biobank and consisted of fresh-frozen astrocytoma tissue obtained intraoperatively. Tissue preservation followed standardized rapid-freezing protocols to maintain molecular integrity. Genomic DNA was extracted using the QIAamp DNA Mini Kit (Qiagen), and its concentration and purity were assessed using the Qubit dsDNA HS Assay Kit (Invitrogen). DNA integrity was verified by 1% agarose gel electrophoresis prior to library construction. DNA quality was assessed using Qubit dsDNA BR Assay and agarose gel electrophoresis to confirm integrity prior to WES. For seizure classification, patients in the non-seizure group were defined as those with no history of clinical seizures prior to surgery, as determined by retrospective review of medical records and neurology consultation. Routine preoperative EEG was not performed in asymptomatic patients, which is consistent with standard clinical practice at our institution.

Written informed consent was obtained from all patients prior to their recruitment. The study received ethical approval from the Human Research Ethics Committee of the Faculty of Medicine, Prince of Songkla University (REC. 65-168-14-1), dated November 8, 2020.

### 2.3 Statistical analyses

Continuous variables (e.g., age, Karnofsky Performance Status [KPS], and tumor volume) were assessed for normality using the Shapiro–Wilk test. Variables that followed a normal distribution were analyzed using independent-samples t-tests, while those with non-normal distributions were analyzed using the Mann–Whitney U test. Categorical variables were assessed using Fisher’s exact test.

To adjust for multiple comparisons in gene-level mutation analysis (n = 142), the Benjamini–Hochberg false discovery rate (FDR) correction was applied. Both raw and adjusted p-values (q-values) are reported for selected gene groups. All statistical analyses were performed using R version 4.2.1, with a significance level set at p < 0.05.

The somatic mutational landscape was visualized using the maftools package. Candidate gene mutations associated with seizures were determined using Fisher’s exact test to compare the proportion of affected patients with somatic mutations in each gene between the two groups. Each group of glutamate receptor gene mutations were also compared with the two groups. A p-value of less than 0.05 was considered statistically significant. All statistical analyses were conducted using R version 4.2.1, with a significance threshold of p < 0.05.

## 3 Results

### 3.1 Basic clinical characteristics and astrocytoma associated seizure

Thirty-four patients with astrocytomas were included in this study, of whom 14 experienced seizures and 20 did not. The groups with and without seizures had mean ages (SD) of 26.9 (7.8) and 30.6 (9.9) years, respectively. Histological diagnoses included 26 patients with glioblastoma, IDH-wildtype, 8 with IDH-mutant (3 astrocytoma, IDH-mutant, grade 2; 2 astrocytoma, IDH-mutant, grade 3; 3 astrocytoma, IDH-mutant, grade 4). The characteristics of the seizures were focal awareness in 3 patients, focal awareness impairment in 1, and 10 had focal-to-bilateral tonic-clonic seizures. Among the non-seizure patients, 10 had tumors located at temporal compared with 7 patients in the seizure group. The clinical characteristics of the patients with astrocytomas are summarized in **Table 1**.

**Table 1:** Clinical and pathological characteristics associated with seizure control.

Characteristic	Non seizure group (n=20)	Seizure group (n=14)	*p*-value
Age (mean + SD.; years)	30.6 (9.9)	26.9 (7.8)	0.32
Sex Female Male	15 4	10 4	0.99
WHO classification CNS5 Astrocytoma, IDH-mutant; CNS WHO grade 2 Astrocytoma, IDH-mutant; CNS WHO grade 3 Astrocytoma, IDH-mutant; CNS WHO grade 4 Astrocytoma, IDH-wildtype	1 0 1 18	2 2 2 8	0.13
Duration of seizure (mean: SD.; months)		3.6 (1.9)	–
Seizure type Focal awareness Focal impair awareness Focal to bilateral tonic clonic	0 0 0	3 1 10	–
Karnofsky Performance Scale	87.5 (6.9)	83.9 (8.3)	0.22
Tumor volume (media + IQR; cm^3^) on T2W1	9.8 (3.2)	10.0 (3.2)	0.41
Tumor location Temporal Extratemporal	10 5	7 7	1.00
Tumor side Left Right	11 9	8 6	0.99
**Glutamate receptor mutation ** Inotropic receptor mutation AMPA receptor mutation (n/N) Kainate receptor mutation (n/N) NMDA receptor mutation (n/N) Metabotropic mutation Group 1 mutation (n/N) Group 2 mutation (n/N) Group 3 mutation (n/N)	0 2 4 0 0 1	1 3 9 2 2 5	0.42 0.62 0.01 0.16 0.16 0.06
IDH1 mutation (n/N)	2	6	0.04
ATRX mutation (n/N)	8	5	1.00
BRAF mutation (n/N)	4	3	1.00
xCT mutation (n/N)	0	1	0.42

n: number with mutation

N: total patients in group

Independent-samples t-test used for age and KPS; Mann–Whitney U test used for tumor volume.

Fisher’s exact test used for all categorical variables.

Univariate analysis of the clinical characteristics showed no significant differences between the seizure and non-seizure groups regarding age, sex, Karnofsky performance scale score, tumor volume, tumor location, or tumor side. In addition, we explored the intergroup differences in glioma WHO grades according to the 2021 classification; no significant differences were observed.

### 3.2 Gene mutations and astrocytoma-associated seizures

**Figure 1:**
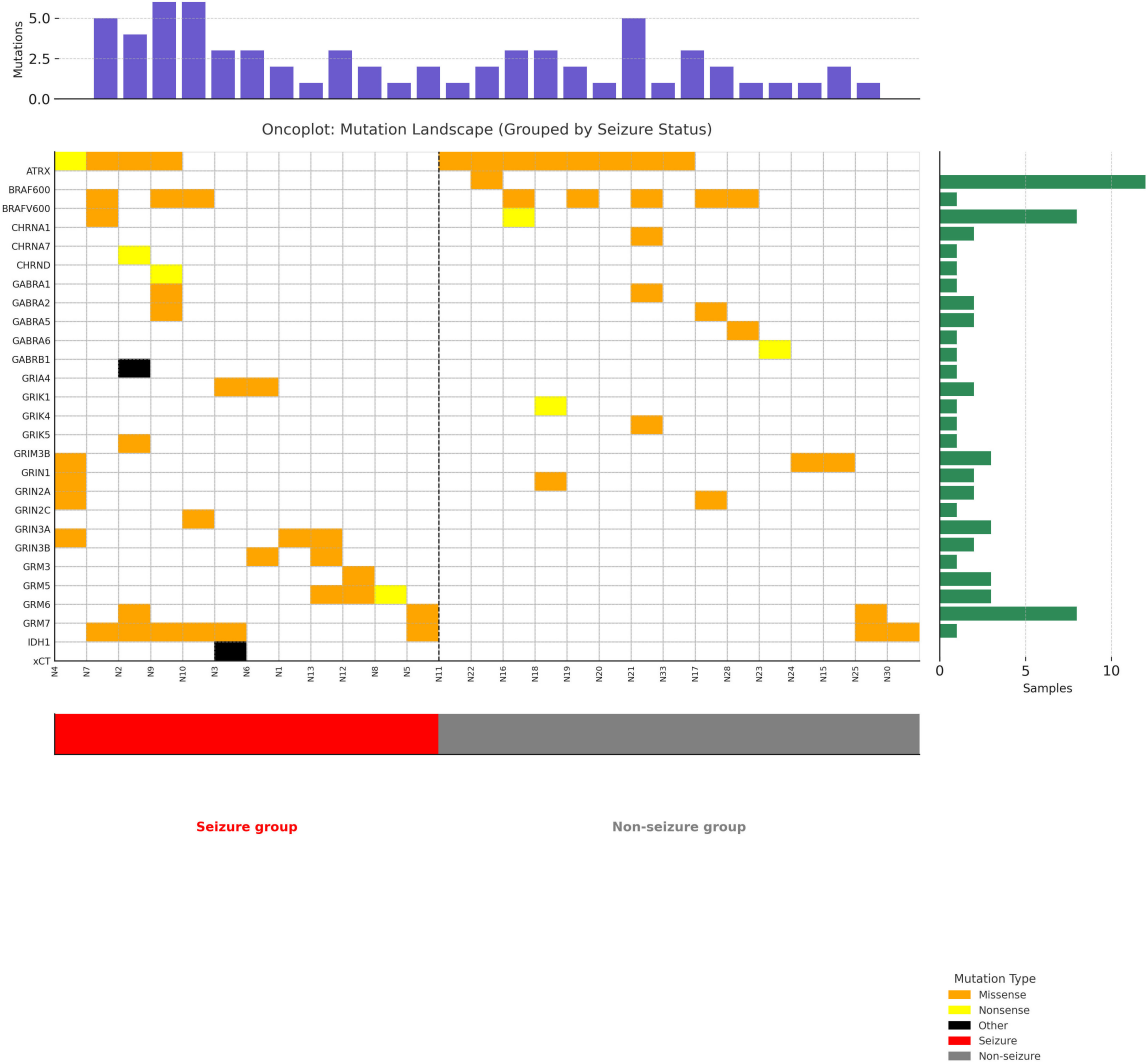
Somatic mutation landscape in patients with Astrocytoma Stratified by seizure status. Oncoplot showing the distribution of somatic mutations across astrocytoma patients, stratified by clinical seizure status. Samples are separated by a dashed vertical line into seizure and non-seizure groups. Mutation types are color-coded (missense, nonsense, other), with upper and side bar plots indicating mutation burden per sample and per gene, respectively. Notably, specific gene subtypes such as IDH1 R132H, BRAF V600E, GRIN2A, and GRIN2C are individually labeled to highlight molecular diversity.

In addition to identifying the candidate genes (see **Supplementary Table 1**), we performed a pathway enrichment analysis using the full gene set. This analysis, summarized in **Supplementary Table 2**, underscores the involvement of glutamatergic and GABAergic synapses, as well as key oncogenic signaling cascades such as PI3K-Akt and MAPK pathways, in astrocytoma-associated seizures. we analyzed mutation frequencies in each functional group and compared them between the seizure and non-seizure cohorts. Exome sequencing of glutamate receptor and signaling genes revealed an *IDH1* mutation in 8/34 individuals and glutamate receptor mutations in 14/34 individuals (11/34 inotropic, 6/34 metabotropic). In the glutamate receptor subgroup, NMDA receptor mutations were detected in 4/20 patients in the non-seizure group and in 9/14 patients in the seizure group (p = 0.01). We found one Group III metabotropic glutamate receptor mutation in the non-seizure group (1/20) but found five mutations in the seizure group (5/14, p = 0.06). *IDH1* mutations, specifically the R132H subtype, were more common in the seizure group (6/14) than in the non-seizure group (2/20) (p = 0.04). Among the seizure patients, 12 out of 14 individuals had multiple gene mutations (**Figure 1**). Statistical comparisons of age, tumor volume, and KPS between the seizure and non-seizure groups were conducted using appropriate tests based on data distribution (i.e., t-test or Mann–Whitney U test). For gene-level mutation analysis (n = 142 genes), we applied the FDR correction to control for multiple testing. Significant or near-significant findings discussed below are based on the FDR-adjusted p-values (q-values). Among the 142 genes screened, 27 genes were found to carry somatic mutations across the cohort. These mutated genes were functionally grouped and summarized in **Supplementary Table 2**. **Figure 1** presents an oncoplot of somatic mutations detected in astrocytoma patients, grouped by clinical seizure presentation. Samples with seizures (red) and without seizures (gray) are visually separated by a dashed vertical line. Mutation types are color-coded, and bar plots indicate mutation burden per sample (top) and mutation frequency per gene (right). Distinct mutational profiles were observed between the groups, with higher frequencies of *IDH1*, *GRIN2B*, and *BRAF* mutations in patients with seizures. This visual distribution further supports the differential mutation burden and potential mechanistic links between glutamatergic signaling and seizure susceptibility in astrocytoma. We did not detect altered expression glutamate signaling genes such as cystine-glutamate transporters, excitatory amino acid transporters 1 and 2 (EAAT1 and EAAT2), BCAT1, or p53 expression. There were no statistically significant differences in GABA receptors, AMPA receptors, kainate receptors, or group 1 and 2 metabotropic glutamate receptors, nicotinic cholinergic receptors, HCAR1, or other gene mutations.

## 4 Discussion

Our results indicate that *NMDA* receptor and *IDH1* gene mutations are significantly associated with patients with astrocytomas presenting with seizures. The occurrence of seizures was not correlated with any clinical characteristics, mutations in other subtypes of glutamate receptor genes, mutations in genes related to glutamate signaling, or other gene mutations (**Table 1**).

The association factors and occurrence of seizures in patients with brain tumors depend on many risk factors, such as age, sex, tumor type, tumor location, tumor size, and histological diagnosis. A large multicenter study including 1509 patients with low-grade glioma found that male sex was the independent factor associated with seizures before operation (19). However, the association between sex and epilepsy in patients with gliomas is not confirmed by other studies (19). Our study did not find the association between sex and seizures in patients with astrocytoma. The association between location of the brain tumor and development of seizures is inconclusive. Some studies have reported temporal or insular location as risks factor for seizure occurrence (1, 19). Feyiseea et al. conducted a retrospective study to identify risk factors for glioblastoma (according to the 2021 WHO classification). GBM-related preoperative seizures were associated with non-occipital tumor location (20). We divided tumor location into two groups (temporal and extratemporal). However, our findings are similar to those of other studies reporting no significant correlation between tumor location and seizure outcomes (8).

Tumor types are the main predictor of seizure development in patients with brain tumors. In a previous study on GRE reported that there was a decrease in epileptogenicity from low-grade to high-grade glioma (6). Low-grade gliomas, including WHO grade II gliomas, consist of astrocytomas, oligodendrogliomas, and oligoastrocytomas and are considered more epileptogenic than high-grade glioma (grade III anaplastic astrocytoma and grade IV glioblastoma) (21). We classified the astrocytoma according to the 2021 WHO classification and did not find an association between the new classification and seizure presentation.

Based on the 2021 WHO CNS tumor classification, multiple molecular characteristics became essential diagnostic criteria for many additional CNS tumor types. Many studies have described the association between gliomas and seizures. Many studies have also reported that various gene mutations can cause excessive glutamate activity and are associated with glioma-associated seizures. In a study of 1,010 diffuse gliomas, *IDH1* mutations were detected in 70.9% of tumors, whereas *IDH2* mutations were observed in only 3.1% of tumors (22). *IDH1* mutations are common in low-grade gliomas (80%) but are rare in primary glioblastomas (4%) (5, 22). Yang et al. reported correlations between *IDH1* mutations and preoperative seizures in patients with grade II and III gliomas (7), but this has not been consistently confirmed (6). *IDH1 R132H*, the most frequent *IDH1* variant, has been previously associated with increased epileptogenicity and favorable prognosis in lower-grade gliomas (23, 24). Using the 2021 WHO classification, we did not find an association between astrocytomas and preoperative seizures (p=0.13).

In contrast, patients with astrocytomas who had recently experienced seizures had significantly higher levels of *IDH1* mutations than those who did not have seizures (6/14 vs. 2/20; *p* = 0.04). However, *IDH2* mutation was not identified in our study. *IDH*-mutant glioma cells reduce α-ketoglutarate to D2HG, which is structurally similar to glutamate and may mimic the activity of glutamate on *NMDA* receptors, potentially leading to seizures (25).

In the past decade, mutations in more than 70 genes have been associated with glioma-associated epilepsy (5, 6). Advances in next-generation sequencing have facilitated comprehensive tumor genome profiling, providing new insights into the somatic mutations and genomic alterations involved in epileptogenesis and tumorigenesis.

Aberrant increases in glutamate activity were found in patients with glioma-associated seizures. Several studies have shown that glutamate receptors are overexpressed in both glioma cells and peritumoral astrocytes (9). However, no study has specifically investigated the association between glutamate receptor mutations and preoperative seizures in patients with gliomas, particularly astrocytomas. In human glioblastomas, human astrocytes express *NMDA* receptors, including GluN2B, which are associated with increased proliferation (26). Increased GluN2B serine (1303) phosphorylation has also been detected in human perigliomal tissue (27). Previous studies suggest that GluN2B-may be related to epileptogenesis in brain tumor-associated epilepsy (9). Indeed, our study showed that NMDA mutations were higher in astrocytoma patients who presented with seizures than in those who did not. In addition, the subtypes of other glutamate receptor mutations did not differ between the two groups. Thus, a larger number of tissues are required to obtain a more comprehensive characterization. Metabotropic glutamate receptors are a family of G-protein-coupled receptors that, with respect to the central nervous system, are predominantly expressed on synapses and are divided into three groups. In our study, we found group III metabotropic glutamate receptor mutations in 1/19 individuals in the non-seizure group and in 5/9 individuals in the seizure group (*p* = 0.06). The functional implications of these mutations in astrocytomas and related seizure activity remain unknown. While important, the sample size in our study was small, and a larger sample is needed to determine the association between this gene and brain tumor-associated epilepsy. For example, xCT has been associated with seizure activity and was previously identified as an independent biomarker for glioma-associated seizures (28). However, in our cohort, the occurrence of seizures was not associated with any xCT gene mutations. Although previous studies have reported SLC7A11 (xCT) and BCAT1 as potential biomarkers for glioma-associated seizures, our dataset did not reveal significant mutation frequencies in these genes. One explanation may be that previous findings focused more on expression levels and transporter activity rather than somatic mutations per se. In particular, recent analyses, such as those by Biegański et al. (29), highlight that dysregulated glutamate homeostasis through xCT overexpression contributes to excitotoxicity and epileptogenesis, which may not be detectable through mutation profiling alone. Therefore, the lack of association in our study might reflect limitations in detecting non-mutational regulatory alterations, and future expression-level or epigenetic analyses are warranted to address this gap.

Conflicting results in patients with gliomas have been reported for other glutamate-related signaling pathways, such as solute carrier family, xCT, BCAT1, PI3K mutation, BRAF mutation, GLT-1, alpha-thalassemia mental retardation X-linked retention, 1p/19q co-deletion, p53 expression, and glutamate receptor genes.

Additionally, other gene mutations, for instance, BRAF variants have been implicated in enhancing epileptogenicity in gliomas (30) while pathways, such as RAS/MAPK and PI3K/AKT/mTOR, play significant roles in both neuronal excitability and tumor growth. Recent multi-omics analyses have revealed that GRE is associated with dysregulation in glutamatergic and GABAergic synaptic pathways. In a comprehensive study involving 997 glioma patients, significant differences were observed in the expression of genes related to synaptic transmission between patients with and without seizures. These findings suggest that alterations in synaptic signaling contribute to the pathogenesis of glioma-associated epilepsy (31).

The most important limitations of this study are its small sample size and the lack of randomization to select the non-seizure group for the paired comparison. Seventy-six percent of our sample had wild-type *IDH* astrocytomas. The underrepresentation of *IDH*-mutant astrocytomas (only 8 of 34 patients, including three with WHO CNS grade 2, two with grade 3, and three with grade 4) limits the generalizability of our findings, particularly to lower-grade glioma populations where seizure incidence is higher. This reflects real-world patterns in tertiary centers, where high-grade gliomas are more often sequenced than lower-grade cases.

This study is primarily intended as a foundational analysis rather than focusing on biomarker identification, and while our analysis identified differentially expressed genes, functional validation studies are necessary to elucidate the precise role of these genes and their associated pathways in astrocytoma-associated epilepsy. In addition, we did not perform RNA-sequencing or methylation. RNA-sequencing provides a comprehensive characterization of the transcriptome, offering insights into the biological processes and pathways involved in tumorigenesis. It helps in understanding the interactions between tumor cells and their microenvironment, which can inform the development of new therapeutic strategies. Based on this knowledge, we plan to conduct transcriptomics and proteomics studies on a larger population.

The strength of our study is that we performed systematic research on patients with astrocytomas based on the 2021 WHO classification that presented with seizures and identified associations with genetic markers of glutamate receptor mutations and other glutamate signaling genes.

## 5 Conclusion

In conclusion, seizures in patients with astrocytomas are associated with *IDH1* and *NMDA* receptor mutations, rather than other clinicopathological factors or other glutamate-related genes. Future large, multicenter studies including functional analyses of *NMDA*-and Group III metabotropic glutamate receptor-related mutations are needed to clarify the exact mechanism of preoperative seizures in patients with astrocytomas.

## Data Availability

The datasets generated and/or analyzed during the current study are available in the SRA repository, which can be accessed via the following link: http://www.ncbi.nlm.nih.gov/bioproject/1223041. The BioProject ID for these datasets is PRJNA1223041.

## References

[1] Chang EF Potts MB Keles GE Lamborn KR Chang SM Barbaro NM. Seizure characteristics and control following resection in 332 patients with low-grade gliomas. J Neurosurg. (2008) 108:227–35. doi: 10.3171/JNS/2008/108/2/0227, PMID: 18240916

[2] Ehara T Ohka F Motomura K Saito R. Epilepsy in patients with gliomas. Neurol Med Chir (Tokyo). (2024) 64:253–60. doi: 10.2176/jns-nmc.2023-0299, PMID: 38839295 PMC11304448

[3] Chen H Judkins J Thomas C Wu M Khoury L Benjamin CG. Mutant IDH1 and seizures in patients with glioma. Neurology. (2017) 88:1805–13. doi: 10.1212/WNL.0000000000003911, PMID: 28404805 PMC5419985

[4] Li H Handsaker B Wysoker A Fennell T Ruan J Homer N. Genome project data processing S. The sequence alignment/map format and SAMtools. Bioinformatics. (2009) 25:2078–9. doi: 10.1093/bioinformatics/btp352, PMID: 19505943 PMC2723002

[5] Rudà R Bruno F Pellerino A. Epilepsy in gliomas: recent insights into risk factors and molecular pathways. Curr Opin Neurol. (2023) 36:557–63. doi: 10.1097/WCO.0000000000001214, PMID: 37865836

[6] Bruno F Pellerino A Conti Nibali M Pronello E Cofano F Rossi M. Association of clinical, tumor, and treatment characteristics with seizure control in patients with IDH1/2-mutant lower-grade glioma. Neurology. (2024) 102:e209352. doi: 10.1212/WNL.0000000000209352, PMID: 38684041

[7] Snijders TJ Berendsen S Seute T Vissers MC Aronica E Robe PA. Glioma-associated epilepsy: Toward mechanism-based treatment. Transl Cancer Res. (2017) 6(Suppl 3):S337–41. doi: 10.21037/tcr.2017.03.03

[8] Jo J Nevel K Sutyla R Smolkin M Lopes MB Schiff D. Predictors of early, recurrent, and intractable seizures in low-grade glioma. Neurooncol Pract. (2020) 8:40–7. doi: 10.1093/nop/npaa054, PMID: 33664968 PMC7906271

[9] Lange F Hörnschemeyer J Kirschstein T. Glutamatergic mechanisms in glioblastoma and tumor-associated epilepsy. Cells. (2021) 10:1226. doi: 10.3390/cells10051226, PMID: 34067762 PMC8156732

[10] Suzuki H Mikuni N Sugita S Aoyama T Yokoyama R Suzuki Y. Molecular aberrations associated with seizure control in diffuse astrocytic and oligodendroglial tumors. Neurol Med Chir (Tokyo). (2020) 60:147–55. doi: 10.2176/nmc.oa.2019-0218, PMID: 32009124 PMC7073702

[11] Wang Z Yang W Wang Y Aili Y Wang Z Wang Q. Correlation of clinicopathological factors with brain tumor-related epilepsy in glioma. Dis Markers. (2022) 2022:4918294. doi: 10.1155/2022/4918294, PMID: 36246555 PMC9553557

[12] Toledo M Sarria-Estrada S Quintana M Maldonado X Martinez-Ricarte F Rodon J. Epileptic features and survival in glioblastomas presenting with seizures. Epilepsy Res. (2017) 130:1–6. doi: 10.1016/j.eplepsyres.2016.12.013, PMID: 28073027

[13] Song L Quan X Chen C Chen L Zhou J. Correlation between tumor molecular markers and perioperative epilepsy in patients with glioma: A systematic review and meta-analysis. Front Neurol. (2021) 12:692751. doi: 10.3389/fneur.2021.692751, PMID: 34539550 PMC8440857

[14] McAfee D Moyer M Queen J Mortazavi A Boddeti U Bachani M. Differential metabolic alterations in IDH1 mutant vs. wildtype glioma cells promote epileptogenesis through distinctive mechanisms. Front Cell Neurosci. (2023) 17:1288918. doi: 10.3389/fncel.2023.1288918, PMID: 38026690 PMC10680369

[15] Li J Long S Zhang Y Wei W Yu S Liu Q. Molecular mechanisms and diagnostic model of glioma-related epilepsy. NPJ Precis Oncol. (2024) 8:223. doi: 10.1038/s41698-024-00721-8, PMID: 39363097 PMC11450052

[16] Fisher RS Cross JH French JA Higurashi N Hirsch E Jansen FE. Operational classification of seizure types by the International League Against Epilepsy: Position Paper of the ILAE Commission for Classification and Terminology. Epilepsia. (2017) 58:522–30. doi: 10.1111/epi.13670, PMID: 28276060

[17] Gritsch S Batchelor TT Gonzalez Castro LN. Diagnostic, therapeutic, and prognostic implications of the 2021 World Health Organization classification of tumors of the central nervous system. Cancer. (2022) 128:47–58. doi: 10.1002/cncr.33918, PMID: 34633681

[18] Mayakonda A Lin DC Assenov Y Plass C Koeffler HP. Maftools: efficient and comprehensive analysis of somatic variants in cancer. Genome Res. (2018) 28:1747–56. doi: 10.1101/gr.239244.118, PMID: 30341162 PMC6211645

[19] Pallud J Audureau E Blonski M Sanai N Bauchet L Fontaine D. Epileptic seizures in diffuse low-grade gliomas in adults. Brain. (2014) 137:449–62. doi: 10.1093/brain/awt345, PMID: 24374407

[20] Feyissa AM Sanchez-Boluarte SS Moniz-Garcia D Chaichana KL Sherman WJ Freund BE. Risk factors for preoperative and postoperative seizures in patients with glioblastoma according to the 2021 World Health Organization classification. Seizure. (2023) 112:26–31. doi: 10.1016/j.seizure.2023.09.013, PMID: 37729723

[21] Kerkhof M Vecht CJ. Seizure characteristics and prognostic factors of gliomas. Epilepsia. (2013) 54:12–7. doi: 10.1111/epi.12437, PMID: 24328866

[22] Nobusawa S Watanabe T Kleihues P Ohgaki H. IDH1 mutations as molecular signature and predictive factor of secondary glioblastomas. Clin Cancer Res. (2009) 15:6002–7. doi: 10.1158/1078-0432.CCR-09-0715, PMID: 19755387

[23] Choate KA Pratt EPS Jennings MJ Winn RJ Mann PB. IDH mutations in glioma: molecular, cellular, diagnostic, and clinical implications. Biology. (2024) 13:885. doi: 10.3390/biology13110885, PMID: 39596840 PMC11592129

[24] Tang T Wang Y Dai Y Liu Q Fan X Cheng Y. IDH1 mutation predicts seizure occurrence and prognosis in lower-grade glioma adults. Pathol Res Pract. (2024) 254:155165. doi: 10.1016/j.prp.2024.155165, PMID: 38286053

[25] Mortazavi A Fayed I Bachani M Dowdy T Jahanipour J Khan A. IDH-mutated gliomas promote epileptogenesis through d-2-hydroxyglutarate-dependent mTOR hyperactivation. Neuro Oncol. (2022) 24:1423–35. doi: 10.1093/neuonc/noac003, PMID: 34994387 PMC9435503

[26] Xing WJ Zou Y Han QL Dong YC Deng ZL Lv XH. Effects of epidermal growth factor receptor and phosphatase and tensin homologue gene expression on the inhibition of U87MG glioblastoma cell proliferation induced by protein kinase inhibitors. Clin Exp Pharmacol Physiol. (2013) 40:13–21. doi: 10.1111/1440-1681.12026, PMID: 23110505

[27] Gao X Wang H Cai S Saadatzadeh MR Hanenberg H Pollok KE. Phosphorylation of NMDA 2B at S1303 in human glioma peritumoral tissue: Implications for glioma epileptogenesis. Neurosurg Focus. (2014) 37:E17. doi: 10.3171/2014.9.FOCUS14485, PMID: 25434386

[28] Sørensen MF Heimisdóttir SB Sørensen MD Mellegaard CS Wohlleben H Kristensen BW. High expression of cystine-glutamate antiporter xCT (SLC7A11) is an independent biomarker for epileptic seizures at diagnosis in glioma. J Neurooncol. (2018) 138:49–53. doi: 10.1007/s11060-018-2785-9, PMID: 29404978

[29] Biegański M Szeliga M. Disrupted glutamate homeostasis as a target for glioma therapy. Pharmacol Rep. (2024) 76:1305–17. doi: 10.1007/s43440-024-00644-y, PMID: 39259492 PMC11582119

[30] Tomschik M Horner E Lang A Mayer F Czech T Kasprian G. BRAF V600E mutation in ganglioglioma: impact on epileptogenicity and implications for surgical strategy. Eur J Neurol. (2025) 32:e70136. doi: 10.1111/ene.70136, PMID: 40186496 PMC11971660

[31] Hartanto RA Dwianingsih EK Panggabean AS Wicaksono AS Dananjoyo K Asmedi A. Seizure in Indonesian glioma patients: associated risk factors and impact on survival. Asian Pac J Cancer Prev. (2021) 22:691–7. doi: 10.31557/APJCP.2021.22.3.691, PMID: 33773530 PMC8286685

