# Supplementary material for: Candidate gene mutations of patients with astrocytoma who present with seizures: evidence from whole exome sequencing
Source: Front Oncol. 2025 Jul 11;15:1577344. doi: 10.3389/fonc.2025.1577344 (PMC12290895; doi:10.3389/fonc.2025.1577344)
Supplement: Supplementary file 1 [file Table1.docx]

**Supplementary Table 1**. Functional Grouping of Genes Screened for Somatic Mutations in Patients with Astrocytoma.

| **Gene** | **Functional Group** |
| --- | --- |
| *CDK4* | Cell Cycle / CDKs |
| *CDK6* | Cell Cycle / CDKs |
| *CDKN2A* | Cell Cycle / CDKs |
| *CDKN2B* | Cell Cycle / CDKs |
| *CHEK2* | Cell Cycle / CDKs |
| *RB1* | Cell Cycle / CDKs |
| *CHEK2* | DNA Repair / Tumor Suppressors |
| *BRCA2* | DNA Repair / Tumor Suppressors |
| *BCOR* | DNA Repair / Tumor Suppressors |
| *TP53* | DNA Repair / Tumor Suppressors |
| *BRCA1* | DNA Repair / Tumor Suppressors |
| *TERT* | Epigenetic Regulators / Chromatin |
| *H3F3A* | Epigenetic Regulators / Chromatin |
| *BCAT1* | Metabolic / Transporters |
| *GRIK2* | Neurotransmission / Receptors |
| *GRIK1* | Neurotransmission / Receptors |
| *GRIA4* | Neurotransmission / Receptors |
| *GRIA3* | Neurotransmission / Receptors |
| *GRIA2* | Neurotransmission / Receptors |
| *GABRQ* | Neurotransmission / Receptors |
| *GABRR3* | Neurotransmission / Receptors |
| *GABRR2* | Neurotransmission / Receptors |
| *GABRR1* | Neurotransmission / Receptors |
| *GRIK4* | Neurotransmission / Receptors |
| *GRIA1* | Neurotransmission / Receptors |
| *GRIK5* | Neurotransmission / Receptors |
| *GRM2* | Neurotransmission / Receptors |
| *GRIN2A* | Neurotransmission / Receptors |
| *GRIN2B* | Neurotransmission / Receptors |
| *GRIN3A* | Neurotransmission / Receptors |
| *GRIN3B* | Neurotransmission / Receptors |
| *GRM1* | Neurotransmission / Receptors |
| *GABRP* | Neurotransmission / Receptors |
| *GRM4* | Neurotransmission / Receptors |
| *GRM5* | Neurotransmission / Receptors |
| *GRM6* | Neurotransmission / Receptors |
| *GRM7* | Neurotransmission / Receptors |
| *GRM8* | Neurotransmission / Receptors |
| *GRIN1* | Neurotransmission / Receptors |
| *GABRG3* | Neurotransmission / Receptors |
| *GRM3* | Neurotransmission / Receptors |
| *GABRG1* | Neurotransmission / Receptors |
| *GABRG2* | Neurotransmission / Receptors |
| *CHRNA1* | Neurotransmission / Receptors |
| *CHRNA10* | Neurotransmission / Receptors |
| *CHRNA2* | Neurotransmission / Receptors |
| *CHRNA4* | Neurotransmission / Receptors |
| *CHRNA5* | Neurotransmission / Receptors |
| *CHRNA6* | Neurotransmission / Receptors |
| *CHRNA7* | Neurotransmission / Receptors |
| *CHRNA9* | Neurotransmission / Receptors |
| *GABRA1* | Neurotransmission / Receptors |
| *CHRNA3* | Neurotransmission / Receptors |
| *GABRA3* | Neurotransmission / Receptors |
| *GABRA4* | Neurotransmission / Receptors |
| *GABRA5* | Neurotransmission / Receptors |
| *GABRA6* | Neurotransmission / Receptors |
| *GABRB1* | Neurotransmission / Receptors |
| *GABRB2* | Neurotransmission / Receptors |
| *GABRB3* | Neurotransmission / Receptors |
| *GABRD* | Neurotransmission / Receptors |
| *GABRA2* | Neurotransmission / Receptors |
| *GABRE* | Neurotransmission / Receptors |
| *NOTCH1* | Others / Unclassified |
| *NF2* | Others / Unclassified |
| *NF1* | Others / Unclassified |
| *MYCN* | Others / Unclassified |
| *MYC* | Others / Unclassified |
| *MET* | Others / Unclassified |
| *MSH2* | Others / Unclassified |
| *MLH1* | Others / Unclassified |
| *MEN1* | Others / Unclassified |
| *MDM4* | Others / Unclassified |
| *NRAS* | Others / Unclassified |
| *MSH6* | Others / Unclassified |
| *PIK3R1* | Others / Unclassified |
| *SMO* | Others / Unclassified |
| *PMS2* | Others / Unclassified |
| *PPM1D* | Others / Unclassified |
| *PTCH1* | Others / Unclassified |
| *PTPN11* | Others / Unclassified |
| *RELA* | Others / Unclassified |
| *RGPD3* | Others / Unclassified |
| *SETD2* | Others / Unclassified |
| *SMARCB1* | Others / Unclassified |
| *SMARCE1* | Others / Unclassified |
| *KRAS* | Others / Unclassified |
| *TRAF7* | Others / Unclassified |
| *TSC1* | Others / Unclassified |
| *TSC2* | Others / Unclassified |
| *USP8* | Others / Unclassified |
| *PLCG1* | Others / Unclassified |
| *KLF4* | Others / Unclassified |
| *FAT1* | Others / Unclassified |
| *IDH* | Others / Unclassified |
| *YAP1* | Others / Unclassified |
| *ATK1* | Others / Unclassified |
| *CHRNB1* | Others / Unclassified |
| *CHRNB2* | Others / Unclassified |
| *CHRNB3* | Others / Unclassified |
| *CHRNB4* | Others / Unclassified |
| *CHRND* | Others / Unclassified |
| *KDR* | Others / Unclassified |
| *CIC* | Others / Unclassified |
| *CTNNB1* | Others / Unclassified |
| *DAXX* | Others / Unclassified |
| *CHRNE* | Others / Unclassified |
| *EAAT1* | Others / Unclassified |
| *HRAS* | Others / Unclassified |
| *DDX3X* | Others / Unclassified |
| *HIST1H3B* | Others / Unclassified |
| *GNAS* | Others / Unclassified |
| *HIST1H3C* | Others / Unclassified |
| *FUBP1* | Others / Unclassified |
| *FGFR3* | Others / Unclassified |
| *FGFR1* | Others / Unclassified |
| *EAAT2* | Others / Unclassified |
| *GNAQ* | Others / Unclassified |
| *xCT* | Others / Unclassified |
| *EGFR* | RTKs / Oncogenic Signaling |
| *PDGFRA* | RTKs / Oncogenic Signaling |
| *KIT* | RTKs / Oncogenic Signaling |
| *BRAF* | RTKs / Oncogenic Signaling |
| *PIK3CA* | RTKs / Oncogenic Signaling |
| *PTEN* | RTKs / Oncogenic Signaling |

This table presents the classification of all genes selected for somatic mutation screening in astrocytoma patients (N = 34). Genes are organized into functional categories based on known biological roles, including cell cycle regulation, DNA repair, epigenetic modification, receptor signaling, neurotransmission, and metabolism. This grouping was used to facilitate interpretation of pathway relevance and molecular mechanisms underlying tumor biology and seizure susceptibility.
