# Supplementary material for: Candidate gene mutations of patients with astrocytoma who present with seizures: evidence from whole exome sequencing
Source: Front Oncol. 2025 Jul 11;15:1577344. doi: 10.3389/fonc.2025.1577344 (PMC12290895; doi:10.3389/fonc.2025.1577344)
Supplement: Supplementary file 2 [file Table2.docx]

**Supplementary Table 2.** Functional Grouping of Genes with Somatic Mutations Identified in Patients with Astrocytoma.

| Gene | Functional Group |
| --- | --- |
| ATRX | Epigenetic / Chromatin Remodeling |
| GABRB1 | GABA Receptors |
| GABRA6 | GABA Receptors |
| GABRA5 | GABA Receptors |
| GABRA1 | GABA Receptors |
| GABRA2 | GABA Receptors |
| GRIK5 | Ionotropic Glutamate Receptors |
| GRIK4 | Ionotropic Glutamate Receptors |
| GRIA4 | Ionotropic Glutamate Receptors |
| GRIK1 | Ionotropic Glutamate Receptors |
| GRM7 | Metabotropic Glutamate Receptors |
| GRM6 | Metabotropic Glutamate Receptors |
| GRM5 | Metabotropic Glutamate Receptors |
| GRM3 | Metabotropic Glutamate Receptors |
| GRIN2C | NMDA Receptors |
| GRIN3B | NMDA Receptors |
| GRIN1 | NMDA Receptors |
| GRIN2A | NMDA Receptors |
| GRIN3A | NMDA Receptors |
| CHRNA7 | Nicotinic Acetylcholine Receptors |
| CHRNA1 | Nicotinic Acetylcholine Receptors |
| CHRND | Nicotinic Acetylcholine Receptors |
| xCT | Transporters / Metabolic |
| BRAF600 | Tumor-related / Oncogenic Drivers |
| BRAFV600 | Tumor-related / Oncogenic Drivers |
| IDH1 | Tumor-related / Oncogenic Drivers |
| GRIM3B | Unknown or Ambiguous |

his table includes genes that were found to carry somatic mutations in the studied astrocytoma cohort (N = 34). Genes are functionally categorized into signaling, neurotransmission, epigenetic regulation, and metabolic transport. The classification reflects the biological relevance of these mutations to tumor pathogenesis and seizure susceptibility.
